# Reserpine improves *Enterobacteriaceae* resistance in chicken intestine via neuro-immunometabolic signaling and MEK1/2 activation

**DOI:** 10.1038/s42003-021-02888-3

**Published:** 2021-12-03

**Authors:** Graham A. J. Redweik, Michael H. Kogut, Ryan J. Arsenault, Mark Lyte, Melha Mellata

**Affiliations:** 1 Department of Food Science and Human Nutrition, Iowa State University, Ames, IA USA; 2 Interdepartmental Microbiology Graduate Program, Iowa State University, Ames, IA USA; 3 Southern Plains Agricultural Research Center, USDA-ARS College Station, TX USA; 4 Department of Animal and Food Sciences, University of Delaware, Newark, DE USA; 5 Department of Veterinary Microbiology and Preventive Medicine, Iowa State University, Ames, IA USA; 6 Present Address: Molecular, Cellular & Developmental Biology, Colorado University-Boulder, Boulder, CO USA

**Keywords:** Mucosal immunology, Agriculture

## Abstract

*Salmonella enterica* persist in the chicken gut by suppressing inflammatory responses via expansion of intestinal regulatory T cells (Tregs). In humans, T cell activation is controlled by neurochemical signaling in Tregs; however, whether similar neuroimmunological signaling occurs in chickens is currently unknown. In this study, we explore the role of the neuroimmunological axis in intestinal *Salmonella* resistance using the drug reserpine, which disrupts intracellular storage of catecholamines like norepinephrine. Following reserpine treatment, norepinephrine release was increased in both ceca explant media and Tregs. Similarly, *Salmonella* killing was greater in reserpine-treated explants, and oral reserpine treatment reduced the level of intestinal *Salmonella* Typhimurium and other *Enterobacteriaceae* in vivo. These antimicrobial responses were linked to an increase in antimicrobial peptide and IL-2 gene expression as well as a decrease in CTLA-4 gene expression. Globally, reserpine treatment led to phosphorylative changes in epidermal growth factor receptor (EGFR), mammalian target of rapamycin (mTOR), and the mitogen-associated protein kinase 2(MEK2). Exogenous norepinephrine treatment alone increased *Salmonella* resistance, and reserpine-induced antimicrobial responses were blocked using beta-adrenergic receptor inhibitors, suggesting norepinephrine signaling is crucial in this mechanism. Furthermore, EGF treatment reversed reserpine-induced antimicrobial responses, whereas mTOR inhibition increased antimicrobial activities, confirming the roles of metabolic signaling in these responses. Finally, MEK1/2 inhibition suppressed reserpine, norepinephrine, and mTOR-induced antimicrobial responses. Overall, this study demonstrates a central role for MEK1/2 activity in reserpine induced neuro-immunometabolic signaling and subsequent antimicrobial responses in the chicken intestine, providing a means of reducing bacterial colonization in chickens to improve food safety.

## Abstract (web summary)

Redweik et al. explore mechanisms underlying the effects of reserpine treatment on *Salmonella*-infected explants from chicken intestine as well as the effects on the intestine in vivo following oral treatment. They demonstrate that several signaling pathways (norepinephrine, mTOR, epidermal growth factor) contribute to these reserpine-induced antimicrobial responses, with MEK1/2 playing a central role.

## Introduction

Poultry products are the primary vehicle for broad-host, nontyphoidal *Salmonella enterica* contamination and foodborne disease in the United States^1,2^, causing 1.35 million infections and costing approximately $400 million annually^3^. Although extensive efforts have been made to minimize *Salmonella* incidence in poultry via antimicrobials, the spread of resistance genes has caused an emergence of *Salmonella* isolates resistant to essential antibiotics^3,4^. Furthermore, live *Salmonella* vaccines and probiotics are commonly implemented as prophylactics in commercial poultry to reduce *Salmonella* load, however, their individual efficacies against *Salmonella* resistance are inconsistent^5–7^. Altogether, current methods are insufficient in the reduction of *Salmonella* in chickens, suggesting that a deeper understanding of biological factors affecting *Salmonella* colonization is needed to develop more successful treatments.

In chickens, broad host *Salmonella* serovars induce an immunotolerant state in the chicken intestine via increased regulatory T cells (Tregs), which suppress the inflammatory immune responses necessary to clear *Salmonella*^8,9^. Thus, interfering with Treg activities in the gut may improve antibacterial responses against *Salmonella*. A largely-understudied field in chicken biology is neuroimmunology, or the interactions between the nervous and immune systems^10^. The intestine is highly-innervated with neurons and immune cell populations, which can then interact via neurochemical signaling^11^. In mammals, Tregs synthesize their own stores of catecholamine neurochemicals like norepinephrine, and disrupting these intracellular stores via reserpine inhibits Treg function^12^. However, whether chicken Tregs have similar neurochemical stores and if they too are affected by reserpine have not yet been investigated.

In this report, we found that reserpine causes the release of intracellular norepinephrine stores from chicken ceca explants and intestinal Tregs, driving increased antimicrobial responses against *Salmonella*. These ex vivo antimicrobial responses were recapitulated in vivo, as birds orally treated with reserpine exhibited reduced gut *Enterobacteriaceae* and *Salmonella* post-challenge compared to control birds. Furthermore, we found that reserpine treatment induced T cell activation, reduced CTLA-4 gene expression, and deactivated metabolic pathways like epidermal growth factor receptor (EGFR) signaling and mammalian target of rapamycin (mTOR) signaling, which were linked to antimicrobial responses. Lastly, we found that MEK1/2 activation plays a central role in reserpine-induced antimicrobial activities.

## Results

### Reserpine treatment induces norepinephrine release from intestinal cells

**Fig. 1:**
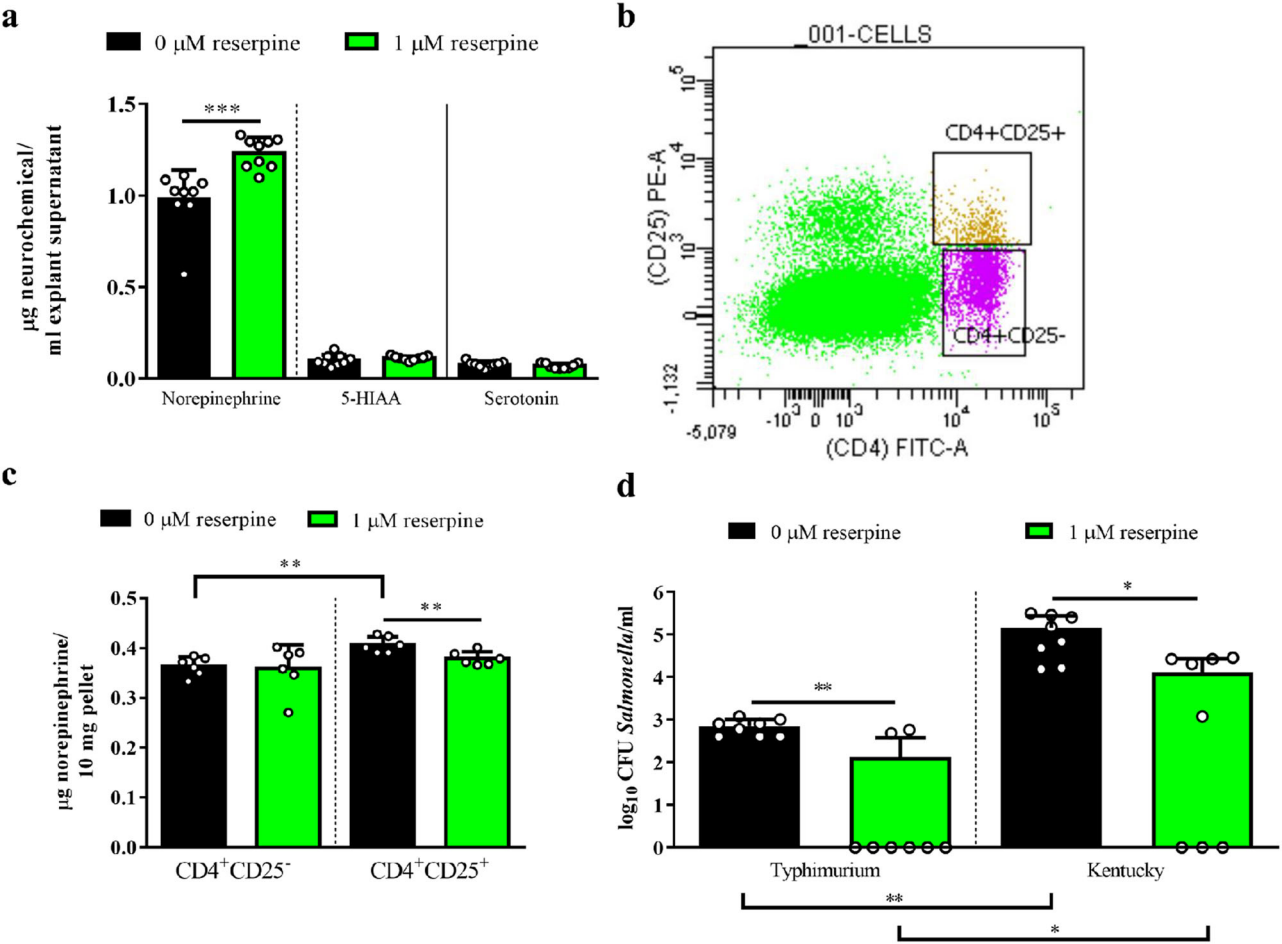
Intracellular norepinephrine release by reserpine increased *Salmonella* resistance ex vivo. Neurochemical release from explants (**a**) and sorted T cells (**b, c**) was evaluated via UHPLC. Reserpine treatment (1 µM) increased bactericidal responses against *Salmonella* in explants (**d**) regardless of serovar. Significant differences indicated by asterisks: *P < 0.05; **P < 0.01; ***P < 0.001. Error bars indicate the standard deviation above and below the mean.

In an intestinal explant model^13^ (Supplementary Fig. 1), we demonstrated neurochemical release in ceca tissues at 1 h post-reserpine treatment (1 µM) using ultra-high-performance liquid chromatography (UHPLC). Culture media from reserpine-treated explants had increased levels of norepinephrine and no changes in serotonergic metabolites compared to controls (Fig. 1a). However, this norepinephrine release did not induce inflammatory damage in the explants, as pathological scores were statistically identical between groups (Supplementary Fig. 2a). Using flow cytometry to sort lymphocyte populations (Fig. 1b) potentially responsible for norepinephrine release in the ceca, Tregs (i.e., CD4^+^CD25^+^) had significantly greater intracellular norepinephrine stores versus naïve T helper (T_H_) cells (i.e., CD4^+^CD25^−^), and reserpine treatment reduced intracellular norepinephrine levels in Tregs alone (Fig. 1c). However, intracellular stores of serotonergic metabolites were unaffected by reserpine treatment (Supplementary Fig. 2b, c).

### Reserpine treatment increases *Salmonella* resistance in ex vivo and in vivo conditions

**Fig. 2:**
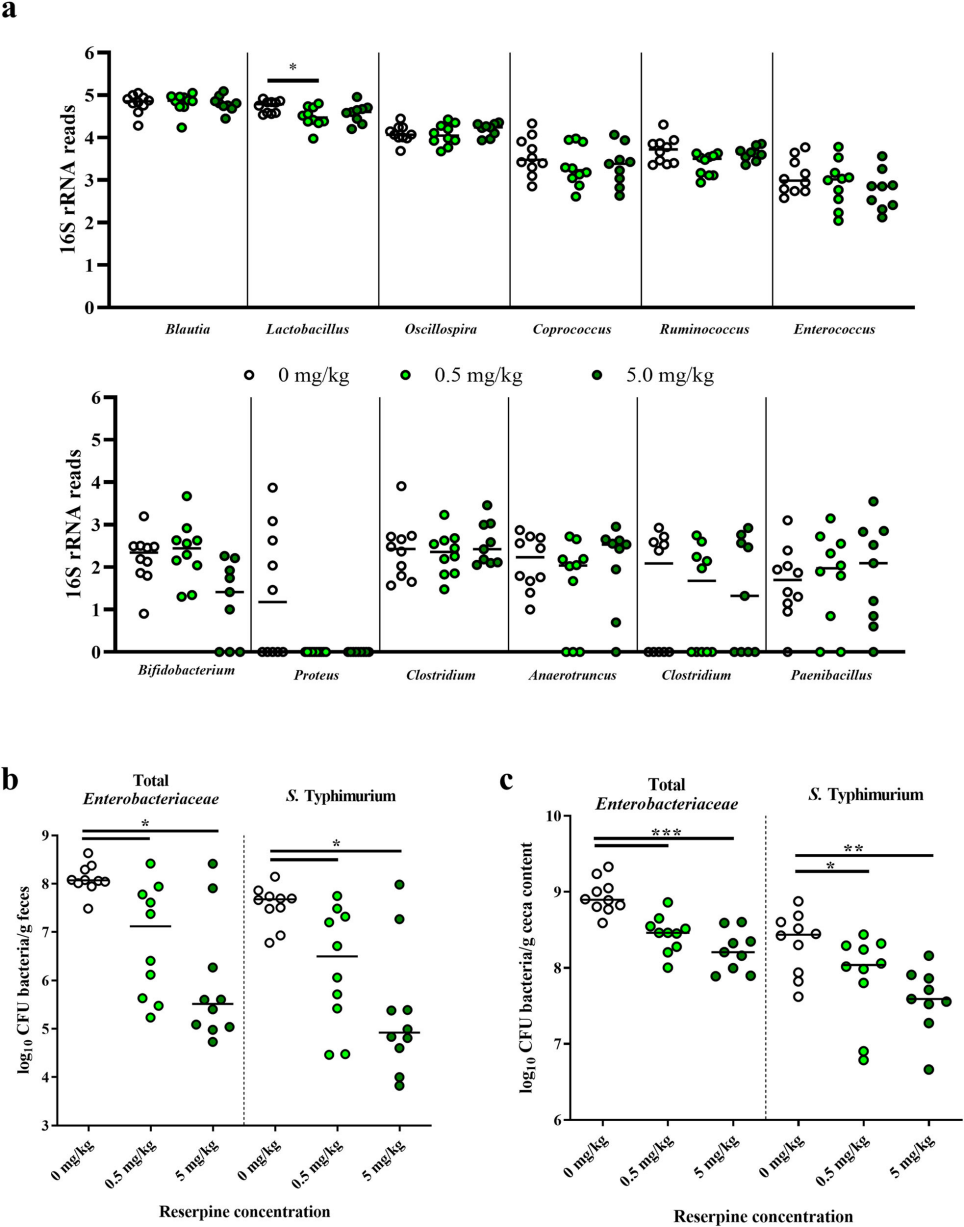
Effect of oral reserpine treatment on commensal and pathogenic bacteria. Oral reserpine treatment did not dramatically affect the composition of the commensal ceca microbiome at the genera level (**a**). However, post-*Salmonella* Typhimurium UK-1 challenge, reserpine treatment reduced total *Enterobacteriaceae* and *S*. Typhimurium UK-1 (**b**, **c**). Significant differences indicated by asterisks: *P < 0.05; **P < 0.01; ***P < 0.001. Error bars indicate the standard deviation above and below the mean.

**Fig. 3:**
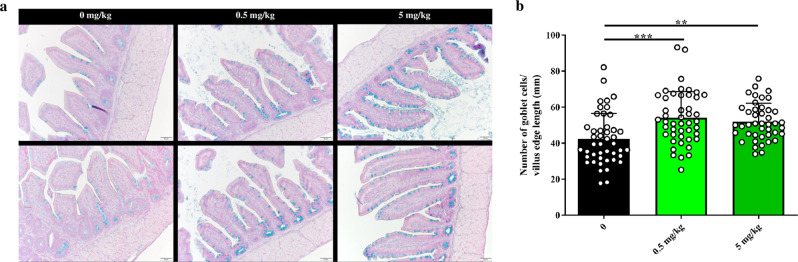
Reserpine treatment increases goblet cell numbers in the chicken ceca. Representative images of Alcian blue staining in ceca tissues (**a**). Total calculations of goblet cells/villus edge length (mm) (**b**). Scale is indicated by white bar (bottom right corner per image; 50 µm). Significant differences indicated by asterisks: **P < 0.01; ***P < 0.001. Error bars indicate the standard deviation above and below the mean.

In ceca explants from 21-day-old birds, supernatant from the reserpine-treated group had higher killing ability against *Salmonella* compared to that of control explants regardless of strains tested, e.g., *Salmonella* Typhimurium and *Salmonella* Kentucky (Fig. 1d). However, reserpine itself was not bactericidal (Supplementary Fig. 2d), confirming that *Salmonella* killing was mediated by host factors. To test in vivo reserpine-induced antimicrobial responses, we orally treated chickens with 0, 0.5, or 5 mg reserpine/kg body weight from 1 to 3 days post-hatch (dph). Reserpine treatment at either concentration did not affect the chicken weight gain at pre- (Supplementary Fig. 3a) nor post-*Salmonella* challenge (Supplementary Fig. 3b), nor did oral reserpine treatment induce the significant release of any neurochemicals systemically (Supplementary Fig. 3c). Given that reserpine induced antimicrobial responses ex vivo, we predicted reserpine may affect the commensal gut microbiota. However, 16S rRNA sequencing showed that reserpine treatment did not affect the levels of the majority of commensal bacteria in the ceca (Fig. 2a and Supplementary Figs. 4, 5). Nevertheless, antimicrobial responses were clearly observed after birds were challenged with *Salmonella* Typhimurium UK-1. At two days post-*Salmonella* challenge, fecal shedding of total *Enterobacteriaceae* and *Salmonella* was significantly reduced by reserpine treatment regardless of concentration (Fig. 2b). Similarly, total *Enterobacteriaceae* and *Salmonella* CFUs in ceca content were reduced by reserpine treatment at four days post-challenge (Fig. 2c). In addition to colonizing the chicken intestine, broad host *Salmonella* strains like UK-1 have the capacity to invade internal organs in young birds^14^. Here, *Salmonella* Typhimurium UK-1 was detected in ceca, spleen, and bursa but not in the liver of challenged birds. Although *Salmonella* levels were statistically identical between groups in the bursa, reserpine treatment significantly reduced *Salmonella* levels in the spleen (Supplementary Fig. 6). Furthermore, reserpine treatment did not induce pathological inflammation at any concentration in the small intestine nor ceca (Supplementary Fig. 7), and ceca goblet cell numbers were significantly increased by reserpine treatment (Fig. 3). This is in line with a previous study demonstrating that, in mammals, reserpine treatment increases the production of intestinal mucus^15,16^, which is synthesized by goblet cells in the epithelium^17^.

### Reserpine treatment increases antimicrobial peptide expression while decreasing CTLA-4 expression

**Fig. 4:**
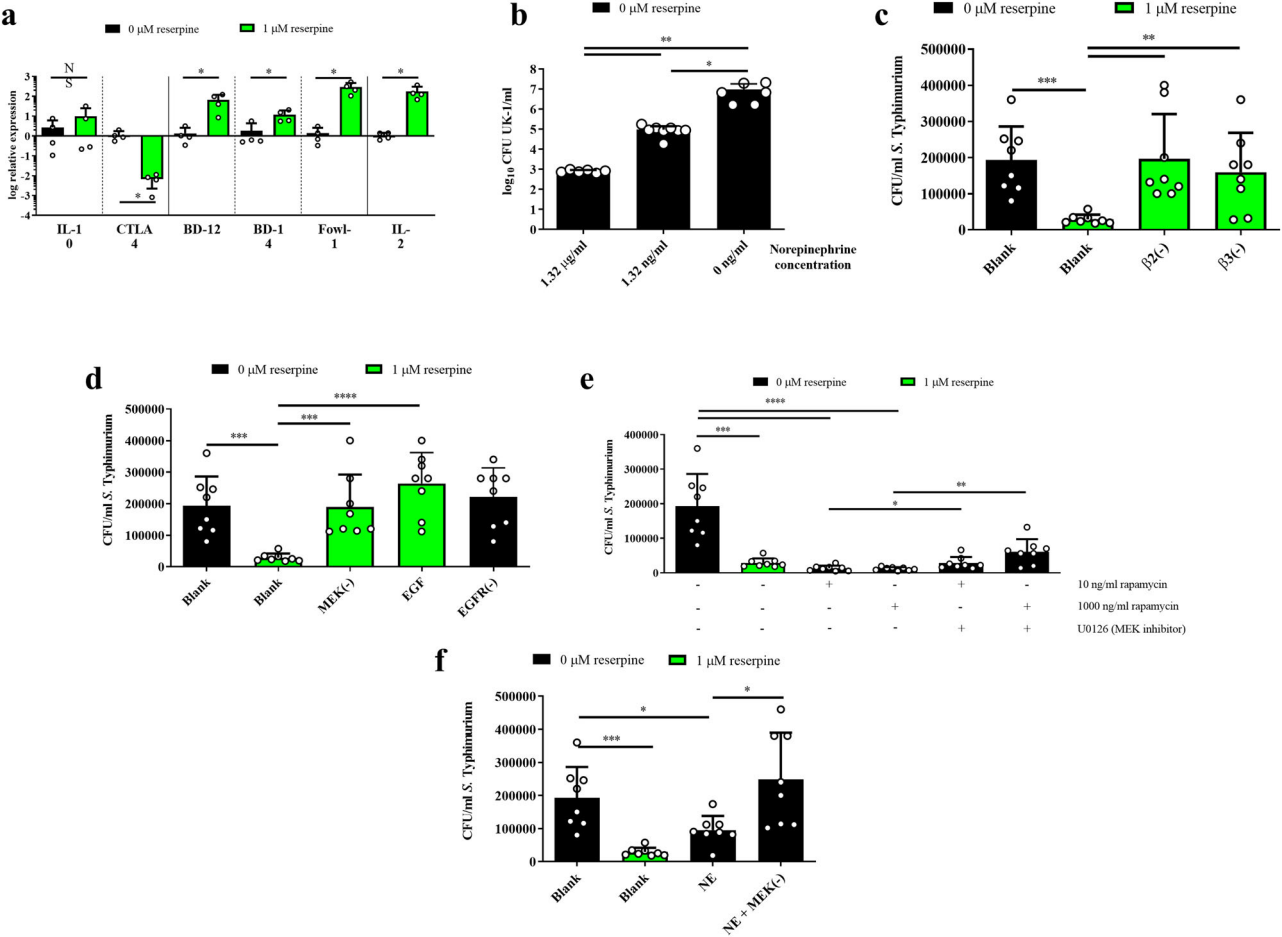
Reserpine treatment increased antimicrobial peptide (AMP) gene expression, and reserpine-induced antibacterial responses were dependent on mTOR, EGFR, and MEK1/2 signaling. AMP and IL-2 gene expression was increased by reserpine treatment while CTLA-4 gene expression was decreased (**a**). Norepinephrine treatment alone increased anti-*Salmonella* responses in explants (**b**), and the effect of reserpine was blocked using beta-adrenergic receptor inhibitors ICI-118551 (*β*_2_) or L-748337 (*β*_3_) (**c**). Reserpine-induced antibacterial activities were inhibited by MEK1/2 kinase inactivation and EGF treatment (**d**), and rapamycin-induced bactericidal responses are partially dependent on MEK1/2 signaling (**e**). Finally, norepinephrine-induced bactericidal responses are dependent on MEK1/2 signaling (**f**). Significant differences indicated by asterisks: *P < 0.05; **P < 0.01; ***P < 0.001; ****P < 0.0001. Error bars indicate the standard deviation above and below the mean.

To determine underlying mechanisms responsible for improved antimicrobial responses upon reserpine treatment, we measured genes expression through transcriptional changes via RT-qPCR. Expression of the regulatory cytokine IL-10^18^ was unchanged (Fig. 4a); however, the expression of CTLA-4, a surface-bound protein associated with Tregs that downregulates immune responses^19^, was downregulated in reserpine-treated explants versus controls (Fig. 4A). In line with this downregulated immunosuppressive factor, reserpine treatment increased gene expression of antimicrobial peptides (AMPs) like beta defensin 12 (BD-12), BD-14, and fowlicidin 1 (Fowl-1) versus controls (Fig. 4a). Furthermore, the expression of IL-2, a cytokine released by activated T cells^20,21^, was also increased by reserpine treatment versus control (Fig. 4a).

### Reserpine-treated explants undergo large immunometabolic shifts

To determine what global immunometabolic pathways were affected by reserpine, we used a chicken-specific kinome peptide array, which measures changes in phosphorylation activities within several signaling pathways^22^. Overall, reserpine treatment altered several immunological and metabolic pathways (Table 1). In total, 414 proteins from the top 25 KEGG pathways were differentially phosphorylated upon reserpine treatment (Table 1). Within these pathways, several were involved in the EGFR signaling pathway and T cell receptor (TCR) signaling pathway, and these pathways were further analyzed. EGFR was dephosphorylated at the Tyr869 residue (Table 2). Furthermore, in the EGFR signaling pathway, mTOR was phosphorylated at Ser2448 and Thr2446 but was dephosphorylated at Ser2481 (Table 2). Uniquely, mitogen-activated protein (MAP) kinase 2 (MEK2), a component of the MEK1/2 signaling pathway^23^, was phosphorylated at the Ser306 residue (Table 2), important for MEK2 activation^24^. Similarly, MEK2 is also involved in the TCR signaling pathway, in which CD28, a T cell co-receptor crucial for T cell activation^25^, was phosphorylated (Table 2).

**Table 1:** Top 25 KEGG pathways in reserpine-treated explants compared to non-treated controls.

KEGG pathway	Observed protein count	False discovery rate
MAPK signaling	54	2.00 × 10^−35^
Insulin signaling	41	3.14 × 10^−34^
Pathways in cancer	63	7.96 × 10^−33^
PI3K-Akt signaling	51	1.22 × 10^−29^
ErbB signaling	29	2.58 × 10^−27^
**EGFR signaling pathway**	**29**	**2.58** **×** **10**^−**27**^
Neurotrophin signaling	32	8.41 × 10^−26^
Focal adhesion	38	8.41 × 10^−−26^
AMPK signaling	32	1.55 × 10^−25^
MicroRNAs in cancer	34	2.76 × 10^−25^
Central carbon metabolism in cancer	26	1.69 × 10^−24^
**T cell receptor signaling**	**29**	**3.38** **×** **10**^−**24**^
Proteoglycans in cancer	35	4.09 × 10^−23^
Insulin resistance	28	2.62 × 10^−22^
Ras signaling	35	3.65 × 10^−21^
HIF-1 signaling pathway	24	1.22 × 10^−18^
Autophagy-animal	26	1.22 × 10^−18^
Regulator of actin cytoskeleton	31	1.22 × 10^−18^
Hepatitis C	26	2.93 × 10^−18^
FoxO signaling	25	2.61 × 10^−17^
Chemokine signaling	28	3.59 × 10^−17^
Toll-like receptor signaling	22	2.97 × 10^−16^
mTOR signaling	25	3.18 × 10^−16^
Adipocytokine signaling	19	8.75 × 10^−16^
B cell signaling	19	1.29 × 10^−15^

The rows in bold indicate the immune or metabolic pathways focused on in this study.

**Table 2:** Phosphorylation status of proteins in the T cell receptor and epidermal growth factor signaling pathways in ceca explants treated with reserpine.

Peptide	Uniprot accession	Phosphorylation site	Fold change	*p*-value
T cell receptor signaling pathway				
PLCG2	P19174	Y783	−1.470	0.00001
RAF1	P04049	S338/S259	1.24191/2.08925	0.00001/0.00016
**MEK2**	**P36507**	**S306/S222**	**1.510/−1.323**	**0/0.0003**
MAP3K8 (TPL2)	P41279	S400/T290	−1.471/−1.26824	0/0.00059
AKT3	Q9Y243	T305	−1.839	0
ZAP70	P43403	Y319	−1.510	0
PAK1	Q13153	T423	1.236	0.0003
NFATC3	Q12968	S344	−1.413	0.0003
c-FOS	P01100	S362	1.195	0.00001
CD28	P10747	Y191	1.286	0.00121
LCK	P06239	Y505	1.116	0.00003
PDPK1	O15530	S241	1.283	0.00003
TAK1	O43318	S439	1.277	0.006
IKK-B	O15111	S180	−1.247	0
JUN	P05412	S63/S73	−1.447/−1.734	0/0
GRB2	P62993	Y209	1.390	0.001
NFATC1	O95644	S269/S245	1.757/1.169	0/0.00161
SOS1	O07889	S1167	1.234	0.00098
h-RAS	P01112	T35	−1.234	0.0001
PTPRC	P08575	Y1216	−1.161	0.005
NF-kB p105	P19838	S337/S932	−1.147/−1.141	0.001/0.009
PI3KR1	P27986	Y476/Y556	1.121/1.114	0.0006/0.014
IL6R	P40189	S782/Y915	−1.169/1.259	0.0142/0.00005
IL7R	P16871	Y449	−1.177	0.0335
IL23R	Q5VWK5	S121	−1.321	0.00007
Il12BR	P29460	Y314	1.175	0.001
SOCS	Q14543	Y221/Y204	−1.303/−1.157	0.0003/0.0026
JAK2	O60674	Y966/Y1007	1.226/−1.260	0.00006/0.0006
JAK1	P23458	Y993/Y1034	−1.384/−1.174	0.002/0.002
STAT1	P42224	Y701	−1.277	0
STAT4	P42228	S722	−1.338	0.002
STAT3	P40763	S727	−1.302	0.0004
Epidermal growth factor receptor signaling pathway				
RPS6KB1	P23445	T412	1.256	0.0003
PLCG1	P10174	Y783	−1.97	0.00001
RAF1	P04049	S338/S258	1.242/2.089	0.0006/0
PDGFRA	P16234	Y1018/Y720	−2.174/−1.135	0.00001/0.01
PDGFRB	P00619	Y579/Y751	−1.414/−1.127	0/0.008
**MEK2**	**P36507**	**S306/S222**	**1.51/1.327**	**0/0.03**
AKT3	Q94243	T305	−1.839	0
KDR	P35968	Y1214	−1.496	0
STAT3	P40763	S727	−1.302	0.0004
**EGFR**	**P00533**	**Y869**	−**1.242**	**0.007**
BRAF	P15056	S729/S446	1.492/−1.338	0/0.0004
PIK3CB	P42338	Y425/S1070	1.636/1/430	0.00001/0.0002
MET (HGFR)	P08581	Y1349/Y1356	−1.180/−1.178	0.01/0.01
GSK3B	P49841	S389	1.161	0.005
FGFR3	P22607	Y760/Y724	−1.258/−1.194	0.015/0.016
EIF4ERP1	Q13541	T37	1.116	0.04
JAK1	P23458	Y993/Y1034	−1.384/−1.174	0.002/0.002
**mTOR**	**P42345**	**S2448/T2446/S2481**	**1.721/1.411/**−**1.672**	**0/0.00001/0.006**
RPTOR	Q8N122	S863	1.245	0.00025
PTEN	P60484	S380/Y240	−1.14/1.247	0.025/0.002
SRC	P12931	S17	1.154	0.004
SHC3	P29353	Y427	−1.208	0.02
JAK2	O60674	Y966/Y1007	1.226/−1.26	0.00006/0.0006
GRB2	P62993	Y209	1.390	0.001
SHC1	P29335	Y262	−1.208	0.02
HRAS	P01112	T35	−1.234	0.0001
PRKCA	P17252	S657/T638	−1.135/−1.204	0.005/0.03
FGFR2	P21802	S782	−1.190	0.02

The phosphorylation status of each significant protein in ceca explant after treatment with reserpine was determined by entering the respective Uniprot accession into phosphorylation site, finding the annotation of the site of interest, and accounting for the phosphorylation fold change (increased or decreased) of that site. Uniprot IDs and phosphorylation sites listed are human orthologs of chicken peptides. Bolded peptides indicate targets of interest in this study.

### Reserpine-induced antimicrobial responses are dependent on norepinephrine and metabolic signaling

Given that reserpine treatment (1) increased intracellular norepinephrine release and (2) induced changes in EGFR and mTOR phosphorylation, we investigated the roles of these pathways in antimicrobial responses. Explants treated with norepinephrine alone similarly induced antibacterial responses in a dose-dependent manner (Fig. 4b), which was blocked by inhibiting beta-adrenergic receptors 2 and 3 (Fig. 4c). Treatment of explants with recombinant EGF alone prevented reserpine-induced antimicrobial responses (Fig. 4d). However, treatment with EGFR inhibitor AG1478 alone did not trigger any antimicrobial responses (Fig. 4d). Additionally, treatment of explants with rapamycin, an inhibitor of the mTOR pathway, increased bactericidal responses (Fig. 4e). Overall, these findings demonstrate that reserpine treatment induces antimicrobial responses through multiple signaling pathways.

### MEK1/2 signaling plays a central role in reserpine-induced antimicrobial responses

In our kinome analyses, we found that these immunometabolic signaling changes were associated with MEK2 phosphorylation, suggesting MEK1/2 signaling plays a vital role in these responses. Using the MEK1/2 signaling inhibitor U0126, MEK1/2 signaling inhibition reversed the antimicrobial response induced by reserpine (Fig. 4d). Similarly, antimicrobial responses in rapamycin-treated explants were partially reversed upon MEK1/2 inhibition (Fig. 4e). Finally, antimicrobial responses in norepinephrine-treated explants were reversed upon MEK1/2 inhibition (Fig. 4f). Overall, these data demonstrate a central role for MEK1/2 signaling in the antimicrobial response induced by reserpine and other neuro-immunometabolic signaling pathways.

## Discussion

Chicken products like meat and eggs are primary vehicles for salmonellosis^1,2^. Reducing *Salmonella* colonization in the chicken intestine is paramount to mitigating salmonellosis in humans. In this study, we demonstrate that reserpine treatment releases intracellular stores of norepinephrine and induces significant changes in chicken ceca immunometabolism, resulting in increased antibacterial responses against *Salmonella*. The ex vivo explant model used in this study allows for preserving the totality of intestinal cell populations present in vivo while maintaining spatial organization, which provides a more accurate representation of in vivo conditions^13^. In support of the utility of this model, we found that reserpine treatment induces antimicrobial responses against *Salmonella* ex vivo and in vivo. In our study, reserpine treatment increased the expression of several AMPs, including beta-defensins 12 and 14 as well as fowlicidin-1. Beta-defensins are crucial to regulating the gut microbiota and homeostasis^26^. Thus, strategies that increase host beta-defensin production are viable replacements for antibiotic treatment^27^. Although these molecules are directly bactericidal, they have additional functions as well. For example, fowlicidin-1 can neutralize bacterial lipopolysaccharide (LPS)^28^, a microbe-associated molecular pattern that potently induces inflammation^29^. Furthermore, beta-defensins reduce intestinal apoptotic signals in LPS-treated animals^30^. Thus, improving the production of these AMPs may both increase resistance against bacterial pathogens, as well as mitigate host damage induced by these antibacterial responses. In support of this, we found no differences in pathological scores between groups despite a clear elevation in immunological responses in reserpine-treated explants. However, the transcriptional factors responsible for reserpine-induced antimicrobial peptide production are unclear at this time. Activation of the transcription factor c-FOS increases antimicrobial responses in macrophages^31^ while suppressing excessive inflammatory responses^32–34^. Given these findings were reflected in our study, reserpine-induced c-FOS activation may be driving these antimicrobial responses, although this remains to be determined.

This reserpine-driven increase in AMP production was associated with increased IL-2 expression and reduced CTLA-4 expression. Upon activation of naïve T cells, IL-2 production is increased, which induces further T cell proliferation, promotes CD4^+^ differentiation, and facilitates effector and memory CD8^+^ T cell formation^20^. This activation process is dependent on the interaction between costimulatory ligand CD28, expressed on naïve T cells, and CD80/86, expressed on antigen-presenting cells (APCs)^35^. However, Tregs can interfere with this interaction via CTLA-4, which outcompetes CD28 for CD80/86 binding, inhibiting IL-2 accumulation and thus preventing T cell activation^25,36^. One of the mechanisms in which *Salmonella* persists in the chicken gut is by increasing intestinal Tregs, which prevents the inflammatory responses necessary to clear *Salmonella*^9^. Thus, we hypothesized that reserpine treatment could inactivate chicken Tregs as shown in human Tregs^12^, which would permit anti-*Salmonella* responses in the gut. As expected, reserpine decreased CTLA-4 expression, which is constitutively expressed on Tregs^37^. We found that CD28 was phosphorylated in reserpine-treated explants, suggesting that CD28 activation and IL-2 production were occurring due to reduced CTLA-4 levels. Furthermore, NFATC1 (but not NFATC2) was phosphorylated upon reserpine treatment. Activation of these transcription factors has been linked to IL-2 production in memory CD4^+^ T cells^38^, suggesting that reserpine is increasing IL-2 gene expression through NFATC1 activation.

One notable observation is that reserpine treatment in vivo did not dramatically change the resident gut microbiota in young birds. The gut microbiota is crucial to proper animal development, driving immune and physiological maturation^39,40^. Furthermore, antibiotic treatment in young animals causes dramatic changes in their gut microbiota^41^, which can predispose them to bacterial infection and physiological dysfunction later in life by depleting populations of gut microbes crucial for normal development^42,43^. Thus, oral reserpine treatment in day-old birds is a feasible way to promote resistance against *Salmonella* without negatively affecting the developing gut microbiota, although the long-term effects of early-age reserpine treatment on the gut microbiota are unclear. Although changes in Fusobacteria*, Lactobacillaceae*, and *Erysipelotrichaceae* were induced by reserpine treatment, these changes were not consistent between reserpine-treated groups and did not appear to be associated with any biological parameter measured in this study (antimicrobial responses, inflammation, mucus production, etc). Thus, the biological impact of these specific shifts in the commensal microbiota is unclear and does not contribute to the host responses investigated in this study. Still, this lack of antimicrobial activity may appear to contrast the reserpine-induced antimicrobial responses seen in our ex vivo explant model. In birds, innately-produced gallinacins are the primary AMPs produced in the intestine at post-hatch, peaking at days 1–3 post-hatch and begin to drop by day 4 post-hatch, in which AMPs controlled through the adaptive immune system become dominant in the chicken intestine^44^. In our study, explants from 21-day-old birds were used to assess reserpine efficacy, in which these intestinal explants would have a more-mature adaptive immune system. Thus, reserpine-induced antimicrobial responses appear to be dependent on the adaptive immune system. This is supported by our finding that reserpine induces norepinephrine release in chicken intestinal Tregs, which coincidentally migrate from the thymus to the chicken intestine around day four post-hatch^45–47^. Dhawan and colleagues (2016) determined that specific subsets of intestinal Tregs are crucial for regulating AMP responses^48^, although the subset of Tregs responsible for this mechanism in chickens is still unclear and warrant further investigation. In humans, reserpine inhibits intracellular vesicle storage of catecholamines such as norepinephrine, which induce autocrine/paracrine signaling loops that suppress Treg function and stimulate immune activation^12^. In this study on chickens, reserpine treatment increased norepinephrine release from both explants and intestinal Tregs. Thus, Tregs at least partially contribute to the total pool of norepinephrine released by intestinal cells. However, in our hands and due to limited reagents and methods for primary chicken cell cultures, we could not culture chicken intestinal Tregs for longer than six hours, preventing any direct examination of reserpine on Treg immunosuppressive function. However, we did find that treatment with norepinephrine alone at the physiological concentration released after one hour of reserpine treatment could stimulate antibacterial responses, which was dependent on beta-adrenergic receptors. Norepinephrine is a well-known mediator of neuroimmunological responses, inducing cytokine production, cell proliferation, and antibody secretion by lymphocytes^49,50^ and has been demonstrated to improve antibacterial responses via cross-talk between sympathetic ganglia and resident tissue macrophages^51^. Overall, the intracellular release of norepinephrine drives antimicrobial responses via autocrine/paracrine signaling of intestinal cell populations. Future work should determine which specific cellular populations (i.e., enterocytes, enteric neurons, APCs) interact with the regulatory T cells involved in this mechanism.

Given the clear immunological stimulation induced by reserpine treatment, we hypothesized that several metabolic pathways might also be affected due to the interplay between host metabolism and the immune system^10^. To this end, we used the chicken kinome peptide array, which measures immunometabolic signaling at the post-translational level^22^ and thus enables a more accurate evaluation of which processes are affected by reserpine. EGFR signaling is crucial for goblet cell-associated antigen passage (GAP) formation in the mammalian intestine^52^, and inhibiting EGFR increases beta-defensin production in intestinal cells in vitro^53^. In this study, we found that EGFR was dephosphorylated in reserpine-treated explants and using recombinant EGF reversed reserpine-induced antimicrobial responses in vitro, demonstrating the importance of EGFR signaling in this system. However, EGFR inhibition alone did not trigger antimicrobial responses, suggesting that EGFR signaling alone is not sufficient to induce antimicrobial responses. Additionally, the mTOR pathway is conserved among eukaryotic organisms and has received vast attention due to its diverse involvement in nutrient sensing, immunity, and aging in animals^54^. Rapamycin, originally derived from the soil bacterium *Streptomyces hygroscopicus*, is commonly used as an mTOR inhibitor^40^. In this study, reserpine induced differential mTOR phosphorylation at multiple sites upon reserpine treatment. Phosphorylation of S2448 and T2446 is carried out by the kinase S6K^55^, and pS2448 drives mTORC1 activation^56^. In this study, mTORC1 may have been activated upon reserpine treatment, as these two mTOR sites, S6K, and raptor (i.e., RPTOR) were all phosphorylated. However, mTORC1 activation does not play a role in these antimicrobial responses, as deactivating mTOR via rapamycin treatment induced similar antimicrobial responses as reserpine treatment. Although these mTOR sites were phosphorylated, S2481 was uniquely dephosphorylated upon reserpine treatment. The sole site for mTOR autophosphorylation^57^, S2481 has been the only site determined to regulate intrinsic mTOR activities^58,59^. Thus, S2481 dephosphorylation deactivates mTOR function, and our study finds that mTOR inhibition increases antimicrobial responses in this ceca explant model. This finding is supported by previous work demonstrating rapamycin treatment increases anti-*Campylobacter* responses in the murine intestine and directly stimulates antimicrobial responses in splenocytes^60^. Thus, in addition to inducing norepinephrine signaling, reserpine also deactivates EGFR and mTOR, and all three of these pathways contribute to antimicrobial responses in chickens. Given that numerous mTOR sites were phosphorylated and dephosphorylated by reserpine treatment, future studies should look at the individual roles of these sites in antimicrobial responses, which could serve as drug targets to promote bacterial resistance.

Although we identified several pathways that differed in phosphorylation patterns, MEK1/2 signaling is well-established as an essential component of beta-defensin production at mucosal barriers^53,61,62^. However, MEK1/2 signaling has never been previously described to be involved in reserpine activity. Here, upon reserpine treatment, MEK2 was phosphorylated at S306. Using the inhibitor U0126, we found that inhibiting MEK1/2 signaling reversed reserpine induced antimicrobial responses, as well as those induced by norepinephrine and rapamycin treatment alone, suggesting that pS306 is a central component of this signaling pathway induced by reserpine and is critical to achieving an antimicrobial response.

In summary, we found that reserpine increases AMP production and immune activation in the chicken intestine by inducing norepinephrine release and beta-adrenergic receptor activation. These changes are correlated with reduced CTLA-4 expression, as well as EGFR and mTOR deactivation, and these antimicrobial responses were dependent on MEK1/2 activation. Thus, we propose that targeting the neuroimmunological axis via oral reserpine treatment could be a viable strategy for increasing *Salmonella* resistance in poultry animals. Furthermore, since oral reserpine treatment also increased resistance against total *Enterobacteriaceae* populations, this treatment may also increase resistance against other bacterial pathogens.

## Materials and methods

### Ethics statement

Animal experiments were approved by Iowa State University Institutional Animal Care and Use Committee, Log # 18-386. Animal enrichments were added to open floor pens to minimize stress during experimental procedures. Euthanasia techniques (CO_2_ asphyxiation) followed the American Veterinary Medical Association Guidelines (2013).

### Ceca explant model and treatment

Methods for chicken ceca explant cultures were adapted from an ex vivo colon explant model for mice^13^ and are summarized in Supplementary Fig. 1. Briefly, 0.1 g tissue pieces from the ceca of 21-day-old chickens were incubated in antibiotic-treated Dulbecco’s modified eagle medium (DMEM) for 30 min at 39.5 °C (5% CO_2_). Explant tissues were then washed with antibiotic-free DMEM to remove residual antibiotics, individually transferred to 24-well plates, and incubated in DMEM with 0 or 1 µM reserpine for six hours at 39.5 °C (5% CO_2_). Alternatively, to confirm reserpine-mediated signaling pathways, tissues were incubated with norepinephrine (1.32 mg/ml or 1.32 µg/ml), beta-adrenergic receptor inhibitors ICI-118551 (*β*_2_; 1 µM; MedChemExpress, LLC) or L-748337 (*β*_3_; 1 µM; R&D Systems), U0126 (MEK1/2 inhibitor; 20 µM; Invivogen), human recombinant EGF (200 ng/ml; Biotang Inc), AG-1478 (EGFR tyrosine kinase inhibitor; 1 µM; BioVision), or rapamycin (mTOR pathway inhibitor; 10 ng/ml or 1000 ng/ml).

### Ultra-high-performance liquid chromatography

To assess neurochemical release from explants, media from explant cultures were centrifuged at 12,000 × *g* for 5 min at 4 °C, and supernatants were pre-treated with 2 M perchloric acid (1:10 dilution), flash-frozen, and stored at −80 °C. Upon thawing, UHPLC with electrochemical detection (UHPLC-EC) was performed on media supernatants as previously described^63^. To assess neurochemical release from lymphocyte populations, regulatory T cells (CD4^+^CD25^+^) or naïve T cells (CD4^+^CD25^−^) were sorted via flow cytometry (see methods section for lymphocyte extraction) and treated with 0 or 1 µM reserpine for 30 min at 39.5 °C. Cells were then pelleted via centrifugation at 300 × *g* for 10 min at 4 °C, and 10 mg pellets were resuspended in 0.2 M perchloric acid, flash-frozen, and stored at −80 °C. Upon thawing, UHPLC-EC was performed on cellular as described earlier.

### Intestinal lymphocyte extraction and flow cytometry

T cells were extracted from the chicken lamina propria as previously described^64,65^. To sort for specific T cell populations, 10^6−7^ cells were resuspended in Zombie violet dye (1:100 solution) and incubated for 20 min at room temperature in the dark. Cells were then centrifuged at 300 × *g* for 5 min at room temperature, and pellets were resuspended via sorting buffer (PBS with 1% FBS) and incubated for 10 min at 4 °C for a blocking step. Thereafter, cells were centrifuged for 5 min at 300 × *g*, and then resuspended with 10 µg/ml anti-CD4 and 10 µg/ml anti-CD25 manually conjugated with Alexa-555 or Alexa-488 fluorophores, respectively. Following a 30 min incubation in the dark at 4 °C, cells were then washed with sorting buffer, and viable CD4^+^CD25^+^ and CD4^+^CD25^−^ populations were sorted via FACSAria III (BD Biosciences) at Iowa State University’s core facility. The gating strategy is exemplified in Supplementary File 1.

### Bactericidal assays against *Salmonella*

Following explant incubation, media from individual explants were centrifuged at 12,000 × *g* for 5 min at 4 °C, and the supernatant was stored at −80 °C until ready for use. *S. enterica* strains (Supplementary Table 1) were grown overnight on LB agar (0.1% glucose), and individual colonies were added to PBS until OD_600_ 0.1. This inoculum was subsequently diluted in PBS until 10^2^ CFU/100 µl was reached. Explant supernatants were added to *Salmonella* inoculum at 1:1 ratio and incubated for six hours at 39.5 °C. Solutions were then serially diluted and plated on MacConkey for bacterial enumeration. All bactericidal assays were run in triplicate.

### In vivo reserpine treatment and *Salmonella* challenge

One-day-old white leghorn chicks (Valo BioMedia, IA) were orally treated daily with 0, 0.5, or 5 mg reserpine per kg body weight (100 µl) for three days. At four days old, chicks were orally inoculated with 100 µl (10^9^ CFU) *Salmonella* Typhimurium strain UK-1 (Supplementary Table 1). Prior to reserpine treatment and *Salmonella* challenge, birds were fasted from food and water for at least 4 h, and food and water were returned to pens 30 min post-treatment and challenge, respectively. Two days post-challenge, feces were serially diluted and plated onto MacConkey agar for *Enterobacteriaceae* and *Salmonella* enumeration. Four days post-challenge, ceca contents, spleen, liver, and bursa were collected from each bird, homogenized, and plated onto MacConkey agar. Chicken weights were collected daily throughout the study.

### DNA isolation and 16S rRNA sequencing

Total DNA was isolated from ceca contents (homogenized from both cecal loops per bird; *n* = 9 or 10 per treatment group) using the DNeasy Powerlyzer PowerSoil Kit (Qiagen). Extracted DNAs were determined for quality via NanoDrop 2000 spectrophotometer (A260/A280). Concentrations were then determined via Qubit dsDNA broad range kit (Thermo Fisher Scientific. DNAs were used for library preparation using the MiSeq and HiSeq2500 kit (Illumina) following all the manufacturer’s instructions with 151 × 151 paired-end MiSeq sequencing (Illumina). 16S rRNA sequencing was performed at the Iowa State DNA facility using Illumina MiSeq (v3). For sequence analysis, using the QIIME2 (version 2019.10) pipeline, sequences were demultiplexed using the demux emp-paired function and denoised using the plugin DADA2. SILVA database at the 99% operational taxonomic units (OTUs) spanning the V4-V5 16S rRNA region (806R: CAAGCAGAAGACGGCATACGAGATAGTCAGCCAGCCGGACTACNVGGGTWTCTAAT; 515F: AATGATACGGCGACCACCGAGATCTACACGCTXXXXXXXXXXXXTATGGTAATTGTGTGYCAGCMGCCGCGGTAA) was used to classify each of the reads using QIIME2’s feature-classifier function. For more details, please refer to the GitHub repository at ISUgenomics/2021_Aug_MelhaMellata_reserpine: reserpine study (github.com). The 16S rRNA dataset is available in the NCBI Sequence Read Archive (SRA) repository with accession BioProject ID PRJNA755726.

### Intestinal pathology scoring and goblet cell enumeration

Explants were placed into 4% paraformaldehyde (PFA) and stored at RT. Subsequently, 5 μm paraffin-embedded cross-sections were stained with hematoxylin and eosin (H&E) to assess gut inflammation. Parameters measuring inflammation (i.e., focal, multifocal, diffuse), infiltrate (i.e., presence of heterophils, lymphocytes, macrophages as well as hemorrhages), necrosis (i.e., focal, multifocal, diffuse), and location (i.e., lamina propria, villous lamina propria, crypt lamina propria) were used. All analyses were performed by a certified pathologist at Iowa State University. To enumerate goblet cells in ceca tissue, sections were stained with Alcian blue to enumerate goblet cells. To quantify goblet cell numbers per villus edge length, the length of the intestinal epithelium was measured using computer software. Goblet cells were then individually counted and divided by villus edge length. Counting on five replicate sections was performed per bird, and 8–10 birds were analyzed per treatment group.

### RT-qPCR

Total RNA was extracted from explant tissues using the PureLink RNA Mini Kit (Life Technologies), and high quality RNAs (A260/A280 ratios ~ 2.0) were assessed via Nanodrop 2000 and quantified via Qubit 2.0 Fluorometer. Reverse transcription assays were performed via High-Capacity cDNA Reverse Transcription Kit (Thermo Fisher) to attain cDNA. Thereafter, SYBR Green (Thermo Scientific) three-step cycling qPCR reactions were performed on StepOnePlus for individual genes (Supplementary Table 2) for 45 cycles. Differences in gene expression were assessed via 2^−ΔΔCt^ method using the housekeeping gene encoding glyceraldehyde 3-phosphate dehydrogenase (GAPDH) as a control^66^.

### Chicken-specific immunometabolic kinome peptide array

Following incubation, ceca explants were flash-frozen, stored at −80 °C, and transported overnight on dry ice to the University of Delaware. Peptide array protocol and analyses were carried out as previously described^22^. The resulting data output was then used in downstream applications such as STRING^67^ and KEGG^54^ databases used to pinpoint changes in the protein−protein interactions and signal transduction pathways.

### Statistics and reproducibility

Statistical comparisons for UHPLC and *Salmonella* resistance data were performed via Student’s t-test or one-way ANOVA on GraphPad Prism software. For the kinome array, signal intensities from scanned array images were arranged into the PIIKA2 input format in Excel, and resultant data were subsequently analyzed via PIIKA2 peptide array analysis software (http://saphire.usask.ca/saphire/piika/index.html). After normalizing these data, we performed comparisons between reserpine-treated and un-treated explants, calculating fold change (= treatment/control) and a significance *P*-value, which was calculated by conducting a one-sided paired t-test between treatment and control values for a given peptide. The resultant fold change and significance values were used to generate optional pathway analysis via standard R statistical functions or online analysis platforms. All in vivo experiments were done in duplicate, and in vitro experiments were performed in triplicate.

### Reporting summary

Further information on research design is available in the Nature Research Reporting Summary linked to this article.

## Supplementary information

Supplementary Information

Description of Additional Supplementary Files

Reporting Summary

Supplementary Data 1

## Data Availability

The 16S rRNA dataset is available in the NCBI Sequence Read Archive (SRA) repository with accession BioProject ID PRJNA755726. Raw kinome data are provided in Supplementary Data 1.
