# Supplementary material for: Reserpine improves Enterobacteriaceae resistance in chicken intestine via neuro-immunometabolic signaling and MEK1/2 activation
Source: Commun Biol. 2021 Dec 3;4:1359. doi: 10.1038/s42003-021-02888-3 (PMC8642538; doi:10.1038/s42003-021-02888-3)
Supplement: Supplementary file 1 — Supplementary Information [file 42003_2021_2888_MOESM1_ESM.pdf]

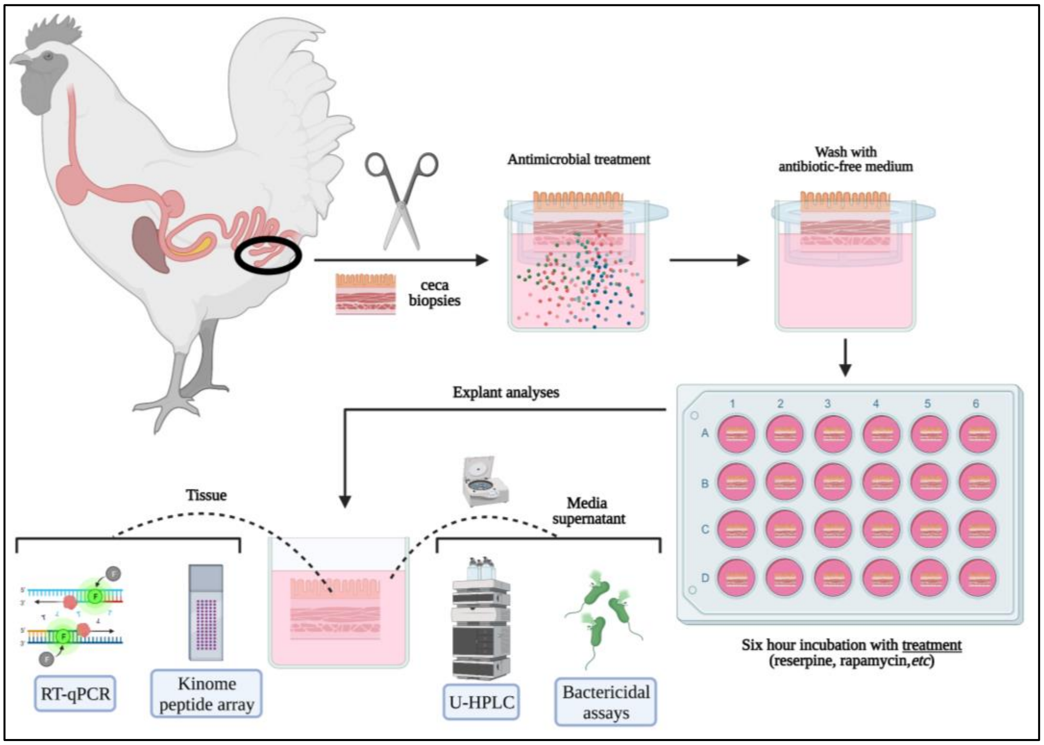

Supplementary Figure 1. Graphical overview of *ex vivo* ceca explant model used in this study. Figure was created using BioRender.com.

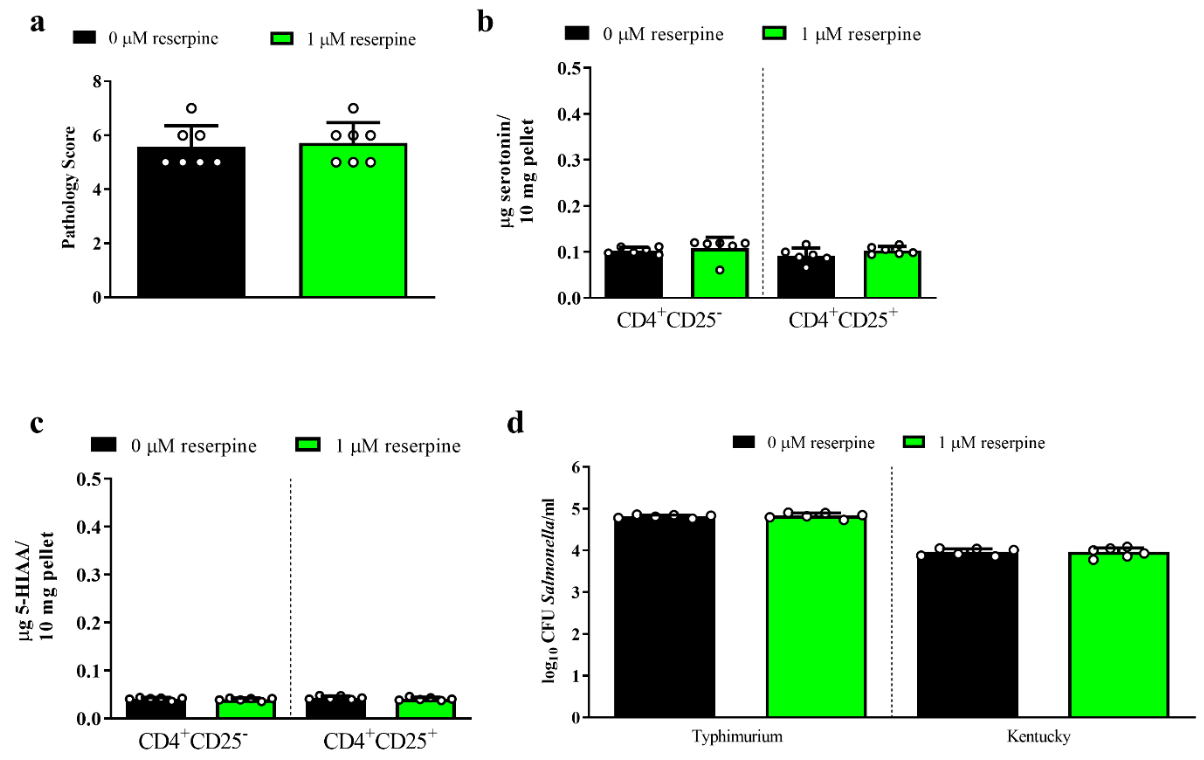

Supplementary Figure 2. a, scoring of explant inflammation via H&E staining. **b**, serotonin concentrations in naïve ($CD4^+CD25^-$) or regulator ($CD4^+CD25^+$) T cell pellets. **c**, 5-hydroxyindolacetic acid (5-HIAA) concentrations in naïve ($CD4^+CD25^-$) or regulator ($CD4^+CD25^+$) T cell pellets. **d**, *Salmonella* bactericidal assays in 0 or 1 µM reserpine-treated media alone. Error bars indicate the standard deviation above and below the mean.

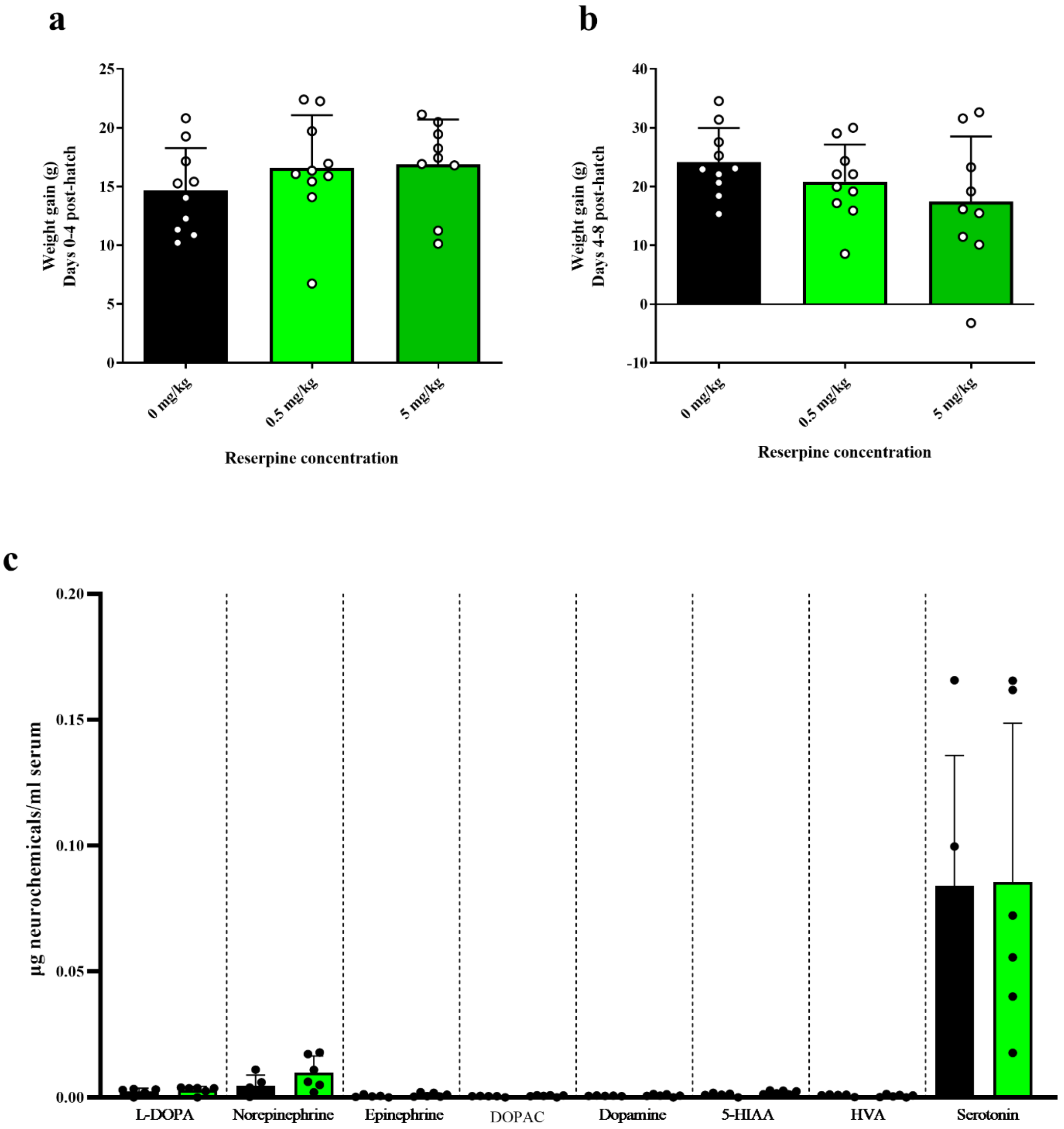

Supplementary Figure 3. a, weight gain (g) of chicks from 0 to 4 days post-hatch (dph). **b**, weight gain (g) of chicks from 4 to 8 dph (*i.e.,* post-*Salmonella* challenge). **c**, neurochemicals detected in serum of chicks 4 dph. Chickens were either not treated (0 mg/kg) or orally-treated with reserpine (0.5 mg/kg bodyweight or 5 mg/kg bodyweight). Error bars indicate the standard deviation above and below the mean.

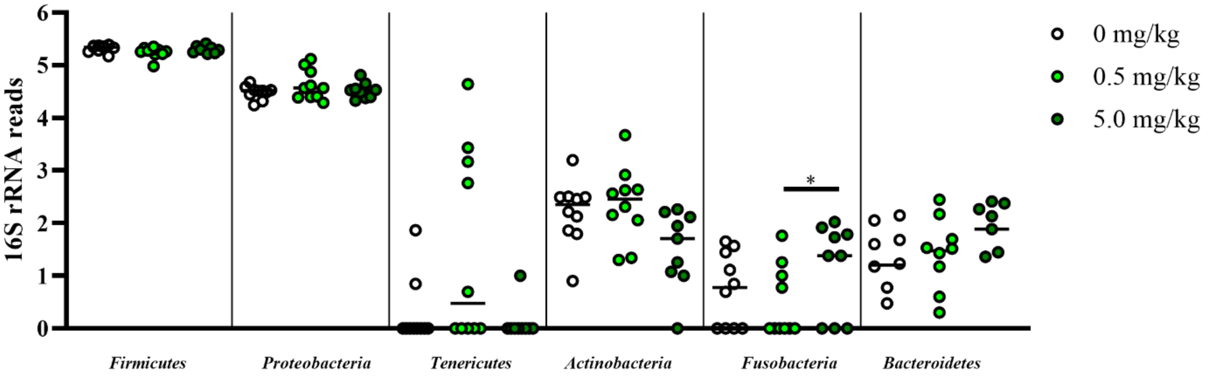

Supplementary Figure 4. 16S rRNA reads of ceca microbiome at phylum level. Chickens were either not treated (0 mg/kg) or orally-treated with reserpine (0.5 mg/kg bodyweight or 5 mg/kg bodyweight). Analyses were performed using QIIME2 pipeline. Error bars indicate the standard deviation above and below the mean.

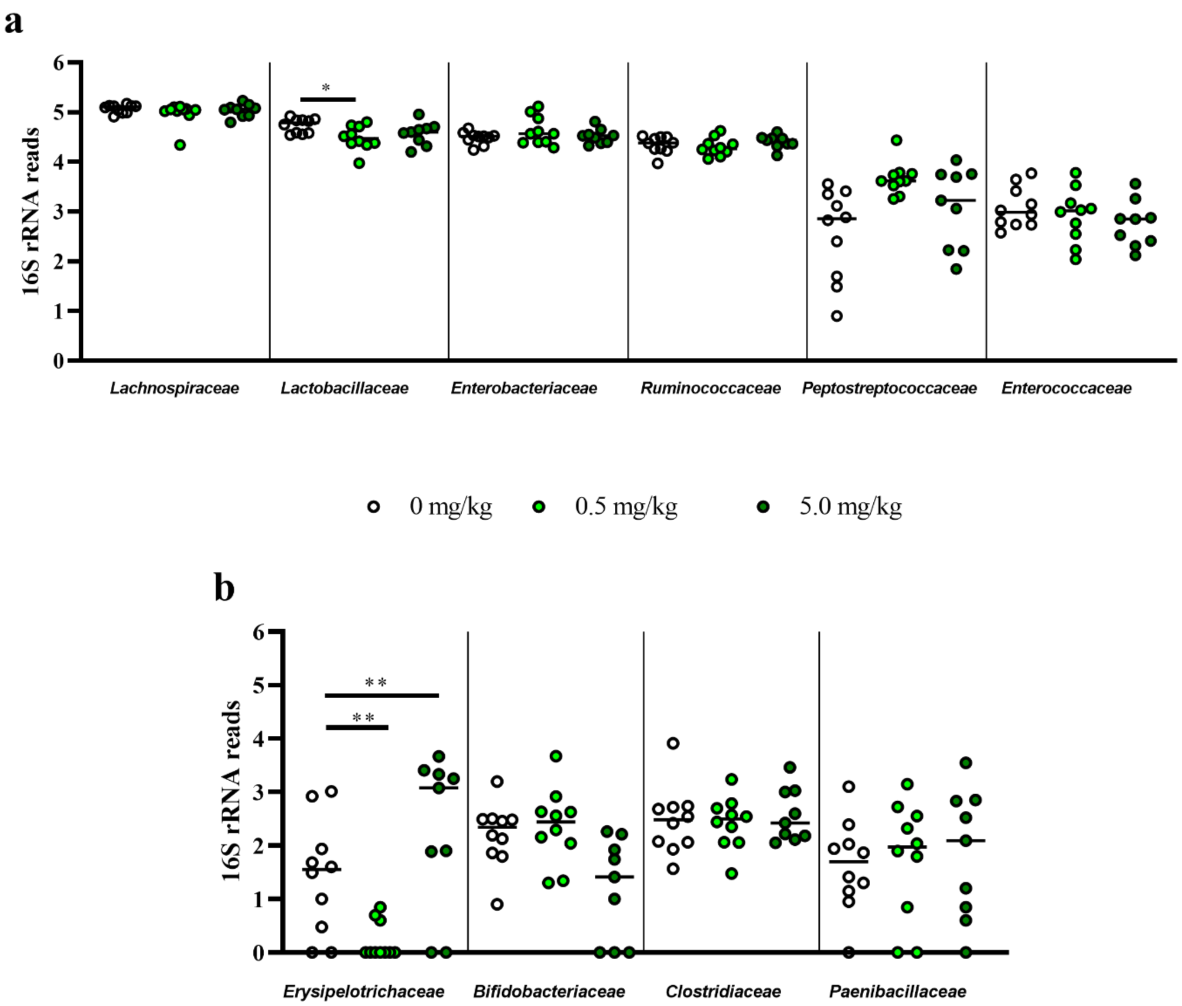

Supplementary Figure 5. 16S rRNA reads of ceca microbiome at family level (a and b). Chickens were either not treated (0 mg/kg) or orally-treated with reserpine (0.5 mg/kg bodyweight or 5 mg/kg bodyweight). Analyses were performed using QIIME2 pipeline. Error bars indicate the standard deviation above and below the mean.

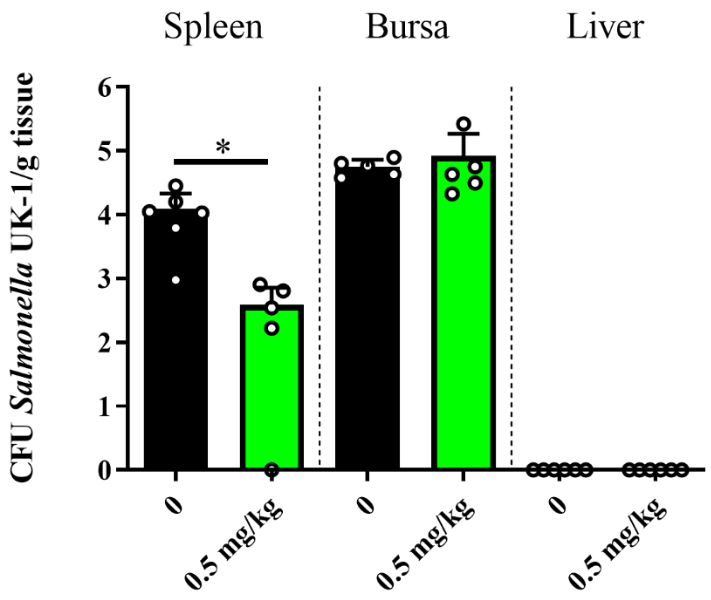

Supplementary Figure 6. Extraintestinal tissue enumeration of *Salmonella* UK-1 two days post-challenge. Chickens were either not treated (0) or orally-treated with reserpine (0.5 mg/kg bodyweight and 5 mg/kg bodyweight). Significant differences indicated by asterisks: *, $P < 0.05$. Error bars indicate the standard deviation above and below the mean.

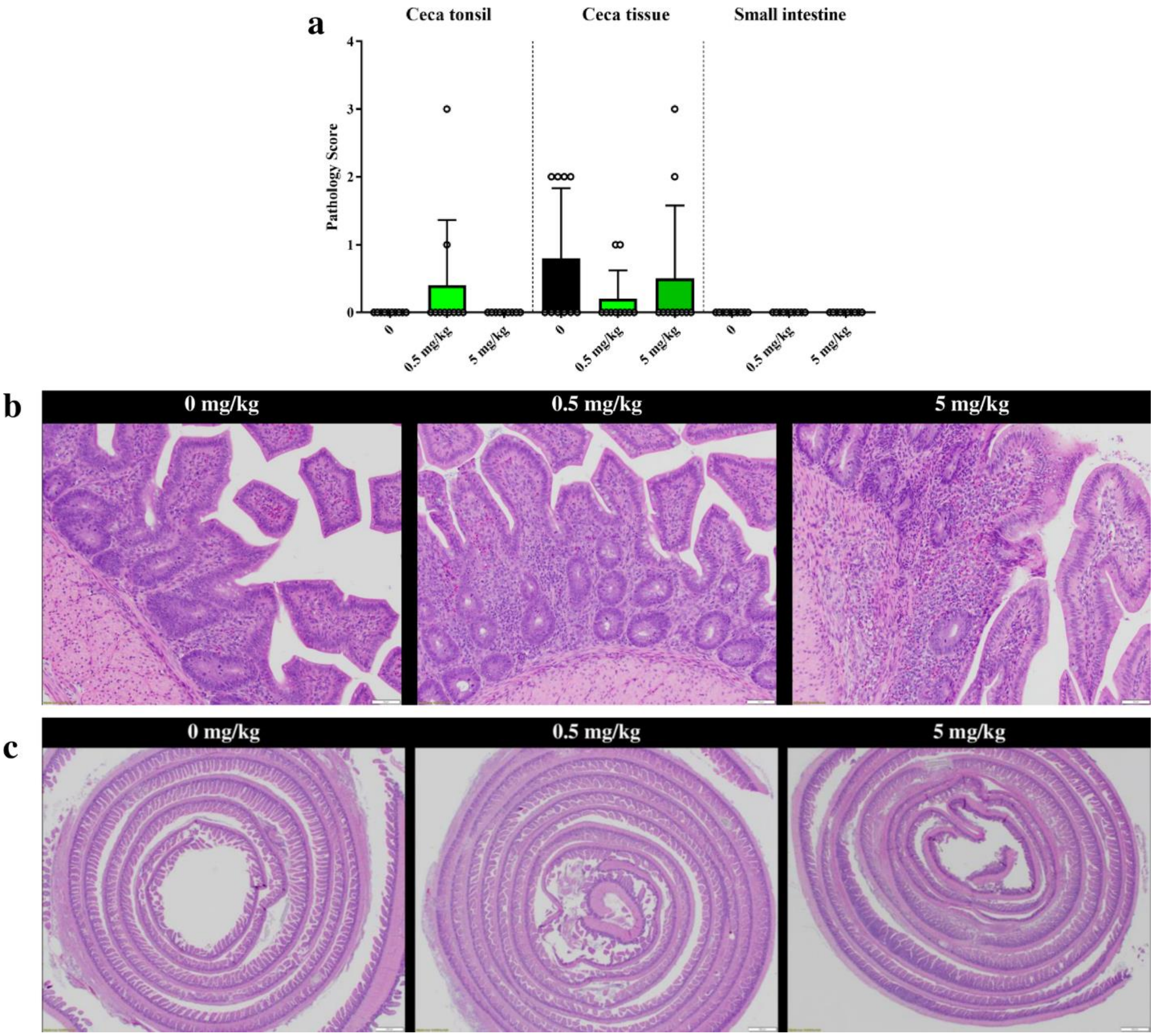

Supplementary Figure 7. a, total scoring of explant inflammation via H&E staining. **b**, representative images of H&E-stained ceca tissues. **c**, representative images of H&E-stained small intestinal swiss rolls. Scale is indicated by white bar (bottom right corner per image): b) 10 µm; c) 100 µm. Error bars indicate the standard deviation above and below the mean.

Supplemental Table 1. Summary of *Salmonella enterica* isolates used for *in vitro* bactericidal assays or *in vivo* challenge. TC, tetracycline. ST, streptomycin. Subscript "R" indicates resistance.

Salmonella enterica (serovar)	Relevant antibiotic-resistance profiles and/or characteristics	Source
**UK-1 (Typhimurium)**	Highly-virulent "universal killer"	(69)
**CVM 29188 (Kentucky)**	TC_R, ST_R, ammonium-resistance	(4)

Supplemental Table 2. Summary of primers and conditions used for qPCR. F, forward primer. R, reverse primer.

Gene Target	Primer sequence (5' – 3')	F/R	Annealing Temp (°C)	Source
**IL-2**	CTGGGAGAAGTGGTTACTCTGA	F	59	(9)
	CCCGTAAGACTCTTGAGGTTC	R		
**IL-10**	CATGCTGCTGGGCCTGAA	F	55	(9)
	CGTCTCCTTGATCTGCTTGATG	R		
**CTLA-4**	CAAGATGGAGCGGATGTACC	F	51	(70)
	TGGCTGAGATGATGATGCTG	R		
**Fowlicidin-1**	GCTGTGGACTCCTACAACCAAC	F	55	(66)
	GGAGTCCACGCAGGTGACATC	R		
**GAPDH**	GCACGCCATCACTATCTTCC	F	55	(66)
	CATCCACCGTCTTCTGTGTG	R		
**Beta-defensin 14**	ATGGGCATATTCCTCCTGT	F	55	(71)
	CACTTTGCCAGTCCATTGT	R		
**Beta-defensin 12**	AGACAGCTGTAACCACGACA	F	55	(71)
	CTGCAGTTCGGACACCTTCA	R		
