# Supplementary material for: Reserpine improves Enterobacteriaceae resistance in chicken intestine via neuro-immunometabolic signaling and MEK1/2 activation
Source: Commun Biol. 2021 Dec 3;4:1359. doi: 10.1038/s42003-021-02888-3 (PMC8642538; doi:10.1038/s42003-021-02888-3)
Supplement: Supplementary file 2 — Description of Additional Supplementary Files [file 42003_2021_2888_MOESM2_ESM.pdf]

Description of Additional Supplementary Files

File name: Supplementary Data 1.
Description: This dataset contains the raw data retrieved from the immunometabolic kinome peptide array and relevant annotations. *P* values < 0.05 are considered significant.
