# Supplementary material for: Reserpine improves Enterobacteriaceae resistance in chicken intestine via neuro-immunometabolic signaling and MEK1/2 activation
Source: Commun Biol. 2021 Dec 3;4:1359. doi: 10.1038/s42003-021-02888-3 (PMC8642538; doi:10.1038/s42003-021-02888-3)
Supplement: Supplementary file 3 — Reporting Summary [file 42003_2021_2888_MOESM3_ESM.pdf]

nature research

Corresponding author(s): Melha Mellata

Last updated by author(s): Oct 11, 2021

Reporting Summary

Nature Research wishes to improve the reproducibility of the work that we publish. This form provides structure for consistency and transparency in reporting. For further information on Nature Research policies, see our Editorial Policies and the Editorial Policy Checklist.

Statistics

For all statistical analyses, confirm that the following items are present in the figure legend, table legend, main text, or Methods section.

n/a	Confirmed	
☐	☒	The exact sample size (*n*) for each experimental group/condition, given as a discrete number and unit of measurement
☐	☒	A statement on whether measurements were taken from distinct samples or whether the same sample was measured repeatedly
☐	☒	The statistical test(s) used AND whether they are one- or two-sided<br>*Only common tests should be described solely by name; describe more complex techniques in the Methods section.*
☐	☒	A description of all covariates tested
☐	☒	A description of any assumptions or corrections, such as tests of normality and adjustment for multiple comparisons
☐	☒	A full description of the statistical parameters including central tendency (e.g. means) or other basic estimates (e.g. regression coefficient) AND variation (e.g. standard deviation) or associated estimates of uncertainty (e.g. confidence intervals)
☒	☐	For null hypothesis testing, the test statistic (e.g. *F*, *t*, *r*) with confidence intervals, effect sizes, degrees of freedom and *P* value noted<br>*Give P values as exact values whenever suitable.*
☒	☐	For Bayesian analysis, information on the choice of priors and Markov chain Monte Carlo settings
☒	☐	For hierarchical and complex designs, identification of the appropriate level for tests and full reporting of outcomes
☒	☐	Estimates of effect sizes (e.g. Cohen's *d*, Pearson's *r*), indicating how they were calculated

Our web collection on statistics for biologists contains articles on many of the points above.

Software and code

Policy information about availability of computer code

Data collection	Raw 16S rRNA sequence reads from chicken ceca digesta DNAs were generated via Illumina MiSeq (v3) at Iowa State University's DNA facility.<br><br>Peptide array data for ceca explants were collected via the chicken specific, immunometabolic kinome peptide array possessed by authors MK and RA.
Data analysis	For sequence analysis of raw 16S rRNA reads, authors used the QIIME2 (version 2019.10) pipeline. Briefly, sequences were demultiplexed using the demux emp-paired function and denoised using the plugin DADA2. SILVA database at the 99% operational taxonomic units (OTUs) spanning the V4-V5 16S rRNA region (806R: CAAGCAGAAGACGGCATACGAGATAGTCAGCCAGCCGGACTAC-NVGGGTWTCTAAT; 515F: AATGATACGGCGACCACCGAGATCTACAC-GCTXXXXXXXXXXXXTATGGTAATTGTGTGYCAGCMGCCGCGGTAA) was used to classify each of the reads using QIIME2’s feature-classifier function. For more details, please refer to the GitHub repository at ISUgenomics/2021_Aug_MelhaMellata_reserpine: reserpine study (github.com).<br><br>For the kinome peptide array analysis, the resulting data output was interpreted in downstream applications such as STRING and KEGG databases to pinpoint changes in the protein–protein interactions and signal transduction pathways.

For manuscripts utilizing custom algorithms or software that are central to the research but not yet described in published literature, software must be made available to editors and reviewers. We strongly encourage code deposition in a community repository (e.g. GitHub). See the Nature Research guidelines for submitting code & software for further information.

Data

Policy information about availability of data

All manuscripts must include a data availability statement. This statement should provide the following information, where applicable:

- Accession codes, unique identifiers, or web links for publicly available datasets
- A list of figures that have associated raw data
- A description of any restrictions on data availability

Kinome peptide array data are available in the Supplemental Data file, whereas

Field-specific reporting

Please select the one below that is the best fit for your research. If you are not sure, read the appropriate sections before making your selection.

☒ Life sciences ☐ Behavioural & social sciences ☐ Ecological, evolutionary & environmental sciences

For a reference copy of the document with all sections, see nature.com/documents/nr-reporting-summary-flat.pdf

Life sciences study design

All studies must disclose on these points even when the disclosure is negative.

Sample size	Sample sizes for in vivo experiments (n = 10 per group) were selected based on pilot studies demonstrating this sample size to be sufficient in demonstrating differences between groups. Similarly, sample sizes for in vitro experiments
Data exclusions	No data were excluded from this study.
Replication	All experiments were performed in duplicate. To exclude pen effect, in vivo experimental treatment groups were duplicated in separate rooms.
Randomization	For explant experiments, ceca explants were pooled post-extraction and then randomly placed into treated or untreated culture wells. For in vivo experiments, animals were randomly placed by animal facility staff into respective pens.
Blinding	The pathologists involved in scoring pathology scores for intestinal tissues were blinded in this study. In addition, U-HPLC (performed in ML lab) and kinome peptide array (performed by MK and RA) experiments were blinded by not disclosing identities of exact treatment groups.

Reporting for specific materials, systems and methods

We require information from authors about some types of materials, experimental systems and methods used in many studies. Here, indicate whether each material, system or method listed is relevant to your study. If you are not sure if a list item applies to your research, read the appropriate section before selecting a response.

Materials & experimental systems

n/a	Involved in the study	
☐	☒	Antibodies
☒	☐	Eukaryotic cell lines
☒	☐	Palaeontology and archaeology
☐	☒	Animals and other organisms
☒	☐	Human research participants
☒	☐	Clinical data
☒	☐	Dual use research of concern

Methods

n/a	Involved in the study	
☒	☐	ChIP-seq
☐	☒	Flow cytometry
☒	☐	MRI-based neuroimaging

Antibodies

Antibodies used	Anti-chicken CD4 (clone CT-4, isotype IgG1; catalogue number 8210-01; Southern Biotech) and anti-chicken CD25 (clone AV142, isotype IgG1; catalogue number MCA5925GA; Bio-Rad) monoclonal antibodies were used for flow cytometry experiments in this study.
Validation	Antibodies were verified in a different study for chicken use (doi: 10.4049/jimmunol.1002040).

Animals and other organisms

Policy information about studies involving animals; ARRIVE guidelines recommended for reporting animal research

Laboratory animals	Gallus gallus, White leghorn chickens
Wild animals	No wild animals were used in this study.
Field-collected samples	No field samples were collected in this study.
Ethics oversight	Animal experiments were approved by Iowa State University Institutional Animal Care and Use Committee, Log # 18-386.

Note that full information on the approval of the study protocol must also be provided in the manuscript.

Flow Cytometry

Plots

Confirm that:

☒ The axis labels state the marker and fluorochrome used (e.g. CD4-FITC).

☒ The axis scales are clearly visible. Include numbers along axes only for bottom left plot of group (a 'group' is an analysis of identical markers).

☒ All plots are contour plots with outliers or pseudocolor plots.

☒ A numerical value for number of cells or percentage (with statistics) is provided.

Methodology

Sample preparation	Lymphocytes were extracted from the ceca lamina propria via chemical (CLSPA liberase, LS005273, Worthington) and mechanical (gentleMACS dissociator; intestine setting), passed through a 70 micron strainer, washed with DNase (100 ug/ml), and resuspended in RPMI prior to labeling with monoclonal antibodies.
Instrument	FACSAria III (BD Biosciences)
Software	BD FACSDiva Version 6.1.3
Cell population abundance	For every extraction, CD4+CD25- cell abundances were approximately 650,000 cells whereas CD4+CD25+ abundances were approximately 200,000 cells (n = 3 birds). Purity was assessed based off gating strategies as described in the next section.
Gating strategy	Live lymphocytes were selected for sorting based on negative staining for Zombie Violet viability indicator, and the typical forward scatter versus side scatter profile of lymphocytes. Live cells are Zombie Violet negative and lymphocytes have relatively low forward and side scatter signatures. Gates for CD4 and CD25 expression on live lymphocytes were established based on fluorescence minus one (FMO) controls. The gate for "positive" CD4-FITC expression was determined based on a sample stained with all reagents except CD4-FITC (page 3 of Supplemental File 1). Conversely, the gate for "positive" CD25-AF555 expression was determined based on a sample stained with all reagents except CD25-PE (page 2 of Supplemental File 1).

☒ Tick this box to confirm that a figure exemplifying the gating strategy is provided in the Supplementary Information.
